# The Frequency of Resistance Genes in *Salmonella enteritidis* Strains Isolated from Cattle

**Published:** 2020-05

**Authors:** Reza RANJBAR, Farhad SAFARPOOR DEHKORDI, Mohammad HEIAT

**Affiliations:** 1. Molecular Biology Research Center, Systems Biology and Poisonings Institute, Baqiyatallah University of Medical Sciences, Tehran, Iran; 2. Halal Research Center of IRI, FDA, Tehran, Iran; 3. Baqiyatallah Research Center for Gastroenterology and Liver Disease, Baqiyatallah University of Medical Sciences, Tehran, Iran

**Keywords:** *Salmonella enteritidis*, Resistance gene, Multi-drug resistance, Cattle

## Abstract

**Background:**

*Salmonella enteritidis* causes infections in humans and animals. Antibiotics are used to eliminate bacterial infections, which become resistant to antibiotics after a while. This study aimed to isolate *S*. *enteritidis* from cattle feces samples and also to evaluate the frequency of genes associated with multi-drug resistance (MDR).

**Methods:**

One hundred ten fecal samples of cattle were collected from Jul to Dec, 2017 in Khuzestan Province, southern Iran. Bacterial culture and molecular methods were used to isolate and identify *S. enteritidis*. Disk diffusion method was used to assess antibiotic susceptibility. Then Polymerase chain reaction (PCR), assay was used for definitive diagnosis of *S*. *enteritidis* and resistance genes.

**Results:**

Overall, 101 (91.81%) samples were detected to be contaminated with *Salmonella* genus and 86 samples (85.14%) were identified as *S. enteritidis*. The highest and lowest antibiotic resistance were belonged to gentamicin (n=70, 81.39%), and tetracycline (n=6, 6.97%). Besides, 64 samples (74.42%) had 2–10 drugs resistance patterns. Moreover, the highest and the least resistance were related to *bla_IMP-1_* (n=73, 84.88%) and *tet(B)* (n=49, 56.97%) genes respectively.

**Conclusion:**

The drug-induced genes in *S*. *enteritidis* have a high frequency. Therefore, antibiotic resistance and high MDR to antibiotics can be due to the incorrect use of antibiotics and the lack of health monitoring in Cattle farms.

## Introduction

*Salmonella* is one of the most important members of the Enterobacteriaceae family. This bacterium is a facultative intercellular, gram-negative, rod-shaped, bacilli-shaped, spore-free, aerobic and anaerobic pathogen (1). Most of the pathogenic *Salmonella* species that cause human disease belong to the *S. enterica* species, which live on the host gastrointestinal tract. *S. enteritidis* (*S*. *enteritidis*) is among the most important disease-causing serotypes. So far, more than 2,500 *Salmonella* serovars have been identified based on flagellar and somatic antigens, most of which are pathogenic to humans and animals (2,3).

*Salmonella* in humans causes diseases such as salmonellosis, intestinal or typhoid fever, septicemia and gastroenteritis. In this regard, *S*. *enteritidis* plays a significant role in the development of these infections (4,5). Diseases transmitted through food are the most important health issues. *Salmonella* is often found in food, meat and dairy products, related to the livestock industry (6,7).

The use of antibiotics for humans and animals causes major health problems, including the presence of antibiotic residues in body tissues and livestock products and the resistance of pathogens to antibiotics, leading to the selection of pathogenic bacteria resistant to multiple drugs (4,8). Multi-drug resistance (MDR) in bacteria is caused by various mechanisms, including reactive changes of the target protein and the enzymatic inactivation of the drug. Enzymatic inactivation is a common resistance mechanism for natural antibiotics such as aminoglycosides and β-lactams (9–11).

Due to increasing rate of MDR bacterium, the purpose of this study was to use PCR as a precise method for detecting *S*. *enteritidis* and also to determine the frequency of resistance genes of this bacterium in cattle samples in Iran.

## Materials and Methods

### Sampling

The present study was conducted from Jul to Dec 2017 in Khuzestan Province, southern Iran. Overall, 110 cattle feces samples were collected randomly and used for detection of *Salmonella*.

### Isolation and Culture of Salmonella

The feces samples were transferred to concentrated peptone water and incubated at 37 °C for 24 h. Then, the samples were transferred to Rappaport Vassiliadis (RV) and incubated again at 43 °C for 24 h. The isolated bacteria were cultured on *Salmonella Shigella* Agar (SSA). Black and gray colonies were considered as suspected *Salmonella*. Complementary assays and differential microbial tests (i.e., IMViC and urease) and culturing on Triple Sugar Iron Agar (TSI) according to Bergey’s manual were used to detect *Salmonella* strain. The lysine decarboxylation was performed on isolated bacteria by culturing in lysine decarboxylase broth to identify the *Salmonella* family. Sulfide Indole Motility medium (SIM) was used for direct detection of *Salmonella* spp. by sulfide production, indole formation, and motility.

### Gene Amplification

Genomic DNA was extracted using QIAamp mini kit (Qiagen, GmbH, Germany). PCR method using specific oligonucleotide primers for *16S rRNA* gene was performed for the direct detection of *Salmonella* family*.* In the next step, *Salmonella*-encoded fimbria (*sefA*) gene was targeted for specific detection of *S*. *enteritidis* from other *Salmonella* strains. The primer sequences used in the present study are shown in Table 1. PCR reactions were performed in a total volume of 25 μL in 0.2 ml tubes containing 2 μL of template DNA, 1 μM of each primer, 2 mM MgCl_2_, 5 μL of 10X PCR buffer AMS, 200 μM dNTPs, and 1 unit of *Taq* DNA polymerase (CinnaClon Co, Iran). The PCR assay was performed at 95 °C for 5 min and then for 32 cycles of 94 °C for 1 min, annealing temp according to Table 1 for 40 sec, 72 °C for 40 sec, and a final extension at 72 °C for 5 min, with a final hold at 10 °C in a thermal cycler (Mastercycler gradient, Eppendorf, Germany). The PCR amplified products were detected in 2% EtBr stained agarose gel electrophoresis.

**Table 1:** Primer sequence of resistance genes and related information in *S*. *enteritidis*

***Resistance Genes***	***Nucleotide sequence (5′ to 3′)***	***Annealing Temperature***	***Size (bp)***	***Reference***
*aadA1*	F:CTCCGCAGTGGATGGCGG	65	311	3
	R:GATCTGCGCGCGAGGCCA			
*aadA2*	F:CATTGAGCGCCATCTGGAAT	65	432	3
	R:ACATTTCGCTCATCGCCGGC			
*aadB*	F:CTAGCTGCGGCAGATGAGC	62	219	3
	R:CTCAGCCGCCTCTGGGCA			
*aadD*	F:TATATCCGTGTCGTTCTGTCCA	55	419	12
	R:CTCTATTTTGCCGATTTATGATTC			
*strA*	F:TGGCAGGAGGAACAGGAGG	62	608	3
	R:AGGTCGATCAGACCCGTGC			
*strB*	F:GCGGACACCTTTTCCAGCCT	65	256	3
	R:TCCGCCATCTGTGCAATGCG			
*bla_TEM-1_*	F:CAGCGGTAAGATCCTTGAGA	55	643	13
	R:ACTCCCCGTCGTGTAGATAA			
*bla_CMY-2_*	F:TGGCCGTTGCCGTTATCTAC	55	870	13
	R:CCCGTTTTATGCACCCATGA			
*bla_IMP-1_*	F:TGAGGCTTACCTAATTGACA	55	324	13
	R:TCAGGCAACCAAACCACTAC			
*bla_CTX-M1_*	F:AACCGTCACGCTGTTGTTAG	55	766	13
	R:TTGAGGCTGGGTGAAGTAAG			
*bla_OXA-1_*	F:AATGGCACCAGATTCAACTT	55	595	13
	R:CTTGGCTTTTATGCTTGATG			
*bla_PSE-1_*	F:TGCTTCGCAACTATGACTAC	55	438	13
	R:AGCCTGTGTTTGAGCTAGAT			
*sul1*	F:TTTCCTGACCCTGCGCTCTAT	55	793	14
	R:GTGCGGACGTAGTCAGCGCCA			
*sul2*	F:CCTGTTTCGTCCGACACAGA	55	667	14
	R:GAAGCGCAGCCGCAATTCAT			
*tet(A)*	F:TTGGCATTCTGCATTCACTC	55	494	14
	R:GTATAGCTTGCCGGAAGTCG			
*tet(B)*	F:CAGTGCTGTTGTGTCATTAA	55	571	14
	R:GCTTGGAATACTGAGTGTAA			
*tet(G)*	F:GCTCGGTGGTATCTCTGCTC	55	550	14
	R:CAAAGCCCCTTGCTTGTTAC			
*cat1*	F:AACCAGACCGTTCAGCTGGAT	55	549	14
	R:CCTGCCACTCATCGCAGTAC			
*cat2*	F:AACGGCATGAACCTGAA	55	547	14
	R:ATCCCAATGGCATCGTAAAG			
*floR*	F:ATGACCACCACACGCCCCG	55	198	14
	R:AGACGACTGGCGACTTCTTCG			
*cmlA*	F:GGCCTCGCTCTTACGTCATC	55	662	14
	R:GCGACACCAATACCCACTAGC			
*sefA*	F:TGCTATTTTGCCCTGTACACTGC	58	214	15
	R:TTCGGGGGAGACTATACCTACAG			
*16s rRNA*	F:AACCGACTCACTCTGGCAG	58	214	15
	R:TAACGCGATAGCGCTTC			

### Antimicrobial drug susceptibility tests

The colonies were cultured on Nutrient Agar (Merck, Darmstadt, Germany) at 37 °C for 24 h after detection of positive *S*. *enteritidis* samples from other *Salmonella* strains. Then, the colonies were sub-cultured into Nutrient Broth (Merck, Germany) and incubated at 37 °C in a shaking incubator for 14 h. McFarland 0.5 turbidity standards were used for bacterial turbidity. After standardization, 100 μL of bacterial suspensions were cultured on Mueller-Hinton agar medium (Merck, Darmstadt, Germany). Drug resistance testing was performed using standard Bauer-Kirby disk diffusion method for all samples (positive culture) according to CLSI protocols for determination of antibiotic susceptibility patterns of *S*. *enteritidis* isolated from feces specimens and the cultures were incubated at 37 °C for 24 h. The antibiotic disks were used for determination of antibiotic susceptibility patterns of *S*. *enteritidis* isolated from feces specimens.

### Detection of antibiotic resistance genes

The PCR was performed for identification of resistance genes. The primers were blasted at the NCBI using the experimental GENINFO BLAST Network Service to assess degree of homology between these primers and other reported sequences and at the end were obtained from CinnaClon Co, Iran. The specific oligonucleotide primers for gene amplification of antimicrobial resistance genes in isolated *S*. *enteritidis* and the sequences of primers are available in Table 1. The PCR was run in final reaction volumes of 25 μL containing the following reagents: 0.2 ml tubes containing 2 μL of template DNA, 1 μM of each primer, 2 mM MgCl_2_, 5 μL of 10X PCR buffer AMS, 200 μM dNTPs, and 1 unit of *Taq* DNA polymerase (CinnaClon Co, Iran). Reactions were initiated at 95 °C for 5 min, followed by 32 cycles of 94 °C for 1 min, annealing temp according to Table 1 for 40 sec, 72 °C for 40 sec and a final extension step at 72 °C for 5 min, with a final hold at 10 °C in a thermal cycler (Mastercycler gradient, Eppendrof, Germany). Detection of PCR products was performed by the method mentioned earlier.

### Statistical Analysis

All statistical tests were performed using Graph Pad Prism statistical software, ver. 7.00 (Graph Pad, San Diego, CA, USA). For all tests, *P*-value <0.05 was considered statistically significant.

## Results

### Identification of bacteria

Out of 110 samples, 101 (91.81%) samples were positive for *Salmonella* by detecting 258 bp band of *16S rRNA* gene. Furthermore, the numbers of 86 (85.14%) specimen were *sefA* gene positive (488 bp).

### Antibiotic susceptibility

**Fig. 1:**
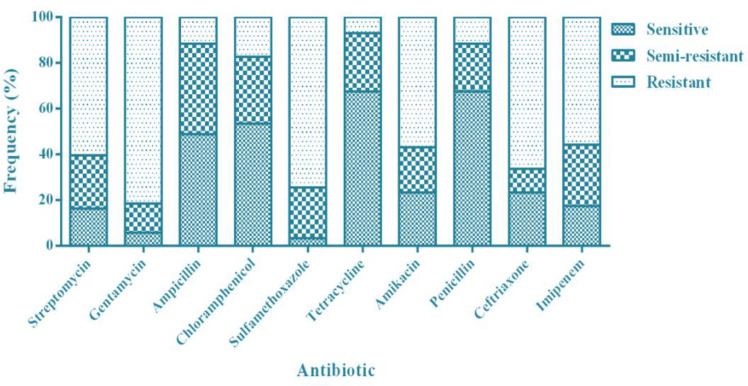
The frequency of antibiotic resistance among the *S*. *enteritidis* under the study

The highest antibiotic resistance was observed to gentamicin (n=70, 81.39%) and the lowest antibiotic resistance was observed in tetracycline (n=6, 6.97%). Moreover, while the lowest antibiotic susceptibility belonged to sulfamethoxazole (n=3, 3.48%) (Fig.1). According to the results, 64 samples had 2–10 drugs resistance patterns (Table 2) in cattle (74.42%).

**Table 2:** The prevalence of MDR in cattle specimens

***NO. of MDR***	***Antibiotics***										***NO. of Animal (n=86)***	***Percent***
0	S	GM	AM	C	SMX	TE	AN	P	CRO	IPM	16	18.60
1	GM										6	6.98
2	GM	SMX									7	8.14
3	GM	SMX	CRO								5	5.81
4	S	GM	SMX	CRO							3	3.49
5	S	GM	SMX	AN	CRO						1	1.16
6	S	GM	SMX	AN	CRO	IPM					33	38.37
7	S	GM	C	SMX	AN	CRO	IPM				5	5.81
9	S	GM	AM	C	SMX	AN	P	CRO	IPM		4	4.65
10	S	GM	AM	C	SMX	TE	AN	P	CRO	IPM	6	6.98

### Resistance genes

In the studied samples, the highest resistance belonged to the *bla_IMP-1_* genes (n=73, 84.88%) and the lowest resistance, on the other hand, was for *tet(B)* (n=49, 56/97%) gene. Detection of resistance genes in isolated *S*. *enteritidis* using PCR technique revealed fragments with specific length sizes in Table 1.

## Discussion

The results of present study revealed that 91.81% and 85.14% were detected for *Salmonella* genus and *S*. *enteritidis* respectively. *bla_IMP-1_* (84.88%) and *tet(B)* (56.97%) genes indicated the highest and the lowest frequency in comparison to other studied genes*.* However, the highest and lowest antibiotic resistances belonged to gentamicin (81.39%), and tetracycline (6.97%) antibiotics.

In Iran, a high prevalence of *S*. *enteritidis* (43%) was reported in comparison with *S. typhimurium*. In contrast, a low *Salmonella* (1.6%) prevalence was reported in Italy (16–18). The frequency of amino-glycoside resistance genes of *aadA1*, *aadA2*, *aadB*, *strA*, and *strB*, of 245 samples were investigated, 62 samples belonged to the *S*. *enteritidis* serotype and the frequency of genes was 45.6%, 34.7%, 31.1%, 37.6%, and 22.4%, respectively (3). By comparing the frequency of genes with the present study, the frequencies are consistent. In Egypt, a study was conducted on 1600 samples to evaluate the multi-drug resistance. The β-lactamases encoding genes were identified in 75.4% of isolates. The frequency of *bla_TEM-1_* gene (41.5%) was relatively high. The highest resistance belonged to ampicillin (95.7%), kanamycin (93.6%), spectinomycin (93.6%), streptomycin (91.5%) and sulfamethoxazole (91.5%) (19). In comparison, in the present study, the highest resistance belonged to gentamicin (81.39%). The frequency of aminoglycoside, tetracycline, sulfonamide, and chloramphenicol genes was investigated and showed the frequency of *sul1* (76.6%), *cat1* (43.3%), *tetA* (40%) and *aadA1* (36.7%) genes (14). Randall et al. conducted some studies on most resistance genes on 397 specimens by culture and molecular methods. They identified resistance to ampicillin (91 isolates), chloramphenicol (85 isolates), gentamicin (2 isolates), streptomycin (119 isolates), tetracycline (108 isolates) and 219 susceptible specimens to all antibiotics (20). In our study, resistance to ampicillin (10 isolates), chloramphenicol (15 isolates), gentamicin (70 isolates), streptomycin (52 isolates) and tetracycline (6 isolates) were identified and 58 isolates were found sensitive to penicillin. Finally, by comparing the two studies, there was a significant relation between the presence of resistance genes and related resistance phenotypes (*P*<0.001).

The phenomenon of antimicrobial resistance has been an urgent global problem since the 1990s (21). The excessive and incorrect use of antimicrobial agents and lack of appropriate infection control are the main possible reasons for this alarming phenomenon and development of MDR in bacteria (22).

In a study, the rate of 52% MDR was shown for Cattle (23). Firoozeh et al. in an Iranian study investigated the prevalence of *S*. enteritidis and the MDR. They identified 77.7% prevalence of *S*. enteritidis, 81% drug resistance, and 69% two or more resistances. The highest resistance phenotypes were streptomycin (58.3%), ampicillin (21.4%), sulfamethoxazole (17.85%), kanamycin (14.28%) and chloramphenicol (14.2%) (24). However, in present study, the highest resistance phenotypes were streptomycin, gentamicin, sulfamethoxazole, amikacin, chloramphenicol and imipenem antibiotics. Due to the presence of streptomycin and sulfamethoxazole antibiotics and the creation of resistance phenotypes in both studies, care should be taken in their use.

## Conclusion

The comparison of the results of present study with others suggests that both the prevalence and the antimicrobial resistance pattern for *Salmonella* is different based on geographical zones. This study determined the widely spread prevalence of *S*. *enteritidis* and MDR in cattle from Khuzestan Province. Efforts that include further implementation of hazard analysis of critical control in livestock production are needed to reduce the incidence of *Salmonella*.

## Ethical considerations

Ethical issues (Including plagiarism, informed consent, misconduct, data fabrication and/or falsification, double publication and/or submission, redundancy, etc.) have been completely observed by the authors.
